# Drug Target Identification and Prioritization for Treatment of Ovine Foot Rot: An* In Silico* Approach

**DOI:** 10.1155/2016/7361361

**Published:** 2016-06-09

**Authors:** Abhishek Acharya, Lalit C. Garg

**Affiliations:** Gene Regulation Laboratory, National Institute of Immunology, Aruna Asaf Ali Marg, New Delhi 110067, India

## Abstract

Ovine foot rot is an infection of the feet of sheep, mainly caused by* Dichelobacter nodosus*. In its virulent form, it is highly contagious and debilitating, causing significant losses in the form of decline in wool growth and quality and poor fertility. Current methods of treatment are ineffective in complete eradication. Effective antibiotic treatment of foot rot is hence necessary to ensure better outcomes during control phases by reduction in culling count and the possibility of carriers of the infection. Using computational approaches, we have identified a set of 297 proteins that are essential to the* D. nodosus* and nonhomologous with sheep proteins. These proteins may be considered as potential vaccine candidates or drug targets for designing antibiotics against the bacterium. This core set of drug targets have been analyzed for pathway annotation to identify 67 proteins involved in unique bacterial pathways. Choke-point analysis on the drug targets identified 138 choke-point proteins, 29 involved in unique bacterial pathways. Subcellular localization was also predicted for each target to identify the ones that are membrane associated or secreted extracellularly. In addition, a total of 13 targets were identified that are common in at least 10 pathogenic bacterial species.

## 1. Introduction

*Dichelobacter nodosus* is a Gram-negative anaerobic bacterium and the main causative organism of ovine foot rot [1]. Foot rot is a contagious and crippling disease affecting the feet of sheep, characterized by a range of symptoms depending on severity, from a nonprogressive interdigital dermatitis (in benign foot rot) to extensive foot infection and separation of hoof from underlying soft tissue, as in case of virulent foot rot [2–4]. The extent of severity depends on the nature of the particular isolate and the climatic conditions. Typically, moist conditions and temperatures above 10°C are essential for transmission [5]. The disease has significantly affected the sheep industry due to morbidity and decline in wool and meat production [6].

The treatment of foot rot generally involves foot-paring and washing with antiseptic solutions such as 10% zinc-sulphate. Foot-paring is generally carried out to remove the diseased tissue and promote healthy foot-structure [4]; however the effectiveness of paring in treatment of foot rot is questionable [7–9] and has been shown to be associated with increased incidences of infection [10]. The application of antibiotics, antibacterial sprays, and solutions has seen much better recovery of affected sheep. Although a number of vaccination programs have been successful in Nepal [11], Bhutan [12], and Australia [13], these are examples where only a single serogroup of* D. nodosus* was infecting the flock. While efforts are underway to develop effective monovalent/bivalent vaccines that can provide adequate cross-protection against multiple strains of* D. nodosus*[14, 15], it is also necessary to develop effective drug-based strategies for the effective treatment of infected flocks.

Large scale sequencing of complete genomes of various pathogens and their hosts has provided a large amount of sequence data at our disposal which could be of much help in identification of drug targets and development of antibiotics. The genomes of* D. nodosus* and the host organism,* Ovis aries*, have been sequenced completely and form the basis of the current* in silico* analysis. Subtractive genomics approach has been used previously for identification of potential drug targets in different pathogenic bacteria such as* Helicobacter pylori* [16],* Pseudomonas aeruginosa* [17, 18],* Mycobacterium tuberculosis* [19],* Aeromonas hydrophila* [20], and* Clostridium perfringens* [21]. Ideally, a drug target should be nonhomologous with host proteins as this would decrease the chances of nonspecific interactions with host proteins and associated side-effects. It is also advantageous if the target protein is known to be “essential” for bacterial survival; any disruption in the functioning of such a protein would lead to death of the bacterial cell. An additional resource that has aided the* in silico* identification of essential genes in pathogenic organisms is the Database of Essential Genes (DEG) [22]. This database contains records for all the essential genes that are currently known and the records are updated as new essential genes are identified and characterized. At present, the DEG consists of essential genes data for 37 pathogenic bacterial species.

In the present work, we performed* in silico* analysis utilizing the BLAST [23] utility and DEG to identify putative drug targets in* D. nodosus*. Further, we have carried out multiple analyses on the list of putative drug targets to classify them on the basis of the pathway/biological process they are involved in and their subcellular localization. Choke-point analyses of the metabolic pathways are a very good method to identify proteins that could be effective drug targets and have been used previously for drug target identification [24–26]. The main objective of this study is to identify prioritized groups of proteins which could be attractive drug targets and can be investigated further using computational and experimental drug discovery methods.

## 2. Materials and Methods

### 2.1. Subtractive Genomics and Prediction of Essential Genes

For the purpose of analysis, complete genome of* D. nodosus* (strain VCS1703A17) and its associated annotation data file were downloaded from NCBI database [14]. Essential genes in* D. nodosus* were predicted by using the Database of Essential Genes (DEG) server [22].* D. nodosus* whole genome sequence along with the annotation data was given as input to the server. The server uses the annotation data to identify the genes and performs BLAST search against DEG. Based on previous studies using similar workflow, an Expectation value cut-off of 10^−10^ and a minimum bit score of 100 were used to shortlist the essential genes [27, 28]. The corresponding protein sequences of all the essential genes were obtained from NCBI and a BLASTP search was performed against a database of sheep protein sequences using an Expectation value cut-off of 10^−4^ for filtering significant hits. Essential genes that were found to be nonhomologous were shortlisted as the putative drug targets. In addition, the results were screened to remove all hypothetical and unknown proteins.

### 2.2. Pathway and Subcellular Localization Analysis of Putative Drug Targets

The putative drug targets that were shortlisted were further analyzed using KAAS (KEGG Automated Annotation Server) to obtain information about the different biological processes and metabolic pathways in which the putative drug targets were involved [29]. This online utility provides rapid and high performance functional annotations of genes by performing BLAST comparison against the KEGG genes database. It automatically assigns *K* number to genes and constructs pathways and BRITE hierarchies.

### 2.3. Choke-Point Analysis

Choke-point analysis of the metabolic pathways of* D. nodosus* was conducted using the BioCyc database which analyzes the pathways information for* D. nodosus* to provide a list of choke-point reactions and the respective protein catalyzing the reaction [30]. The list of potential drug targets obtained for* Dichelobacter* was cross-referenced against this list of choke-point reactions to identify those drug targets that were choke-point proteins in addition to being essential and nonhomologous with host proteins. The results of this analysis were manually cross-checked with KEGG pathways database [31].

### 2.4. Subcellular Localization

PSORTb server was used to predict the subcellular localization of the potential drug targets in order to analyze the distribution of the drug targets into different compartments of the cell [32]. The results were also cross-checked using the CELLO web server [33].

### 2.5. Conservation across Multiple Pathogenic Species

**Figure 1:**
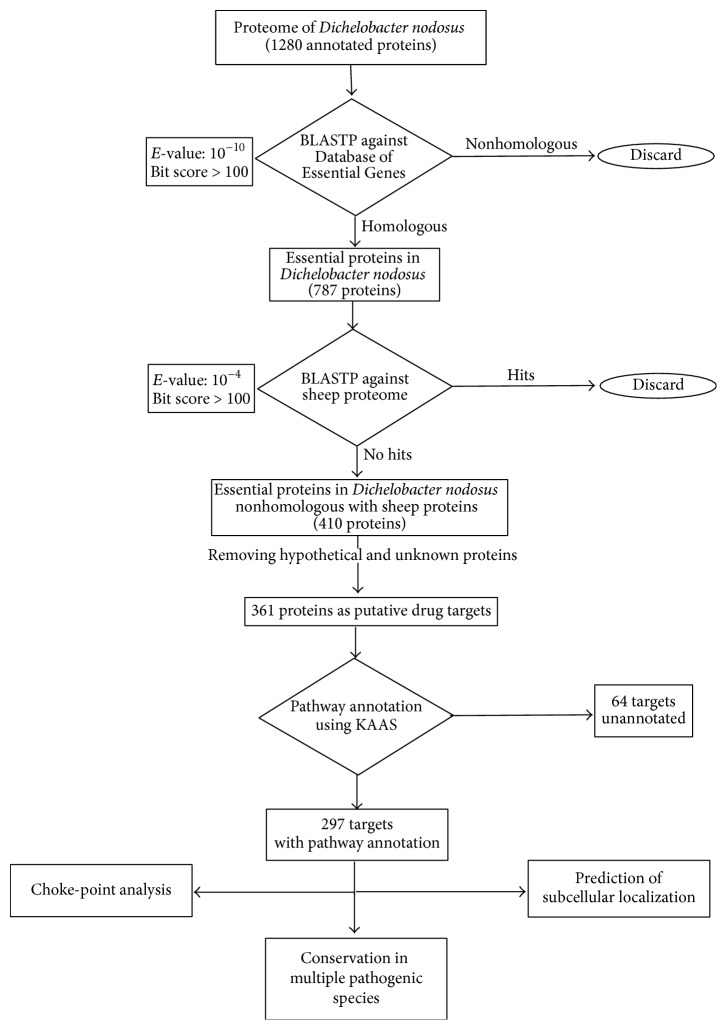
Flowchart depicting the workflow used in this study for identification of putative drug targets and target prioritization against* Dichelobacter nodosus*.

Putative drug targets were analyzed to identify the ones that are also essential to 12 other pathogenic bacterial species, namely,* Helicobacter pylori* 26695,* Acinetobacter baylyi* ADP1,* Haemophilus influenzae* Rd KW20,* Bacillus subtilis* 168,* Mycobacterium tuberculosis* H37Rv,* Staphylococcus aureus* N315,* Campylobacter jejuni* subsp.* jejuni* ATCC 700819,* Francisella novicida* U112,* Salmonella enterica* serovar Typhimurium SL1344,* Mycobacterium tuberculosis* H37RvIII,* Streptococcus pneumoniae*, and* Vibrio cholerae* N16961. BLASTP was performed against the protein sequence database of the aforementioned species present in DEG. An *E*-value of 10^−5^ and a bit score of 100 were used for the analyses. A flowchart of the workflow employed for the present study is depicted in Figure 1.

## 3. Results and Discussion

### 3.1. Subtractive Genomics and Essentiality Prediction for Filtering Drug Targets

*In silico* subtractive genomic analysis is a very fast and efficient method for identifying proteins in pathogenic species that are absent in the host. These proteins could serve as potential drug targets against the pathogens infecting the host tissues. Another important condition is the essentiality of the pathogen-specific proteins. Essential proteins are those which are believed to be critical for the survival of a cell. Although the essentiality of a gene is dependent on specific environment and cellular conditions, in general, the essentiality of a protein target is a positive indicator for druggability of the target. Therefore, we have identified a subset of proteins in* Dichelobacter nodosus* that are both essential to the pathogen and nonhomologous with ovine proteins.

The 1.39 Mb genome of* D. nodosus* VCS1703A is the smallest known genome of an anaerobe, containing 1354 annotated genes that encode for 1280 proteins [14]. BLAST analysis of the genome using DEG server gave a list of 787 protein coding genes that were predicted to be essential for the survival of* D. nodosus*. Thereafter, BLASTP analysis was performed for these 787 protein sequences against the sheep protein sequence database to identify 410 proteins that gave no significant hits; that is, they do not have a significant homology with any of the host proteins. Out of these protein sequences, 49 were hypothetical protein sequences and were not considered for any further analysis. A final list of 361 proteins was obtained that were most likely to be ideal drug targets against* D. nodosus*.

### 3.2. Pathway Annotation of Drug Targets

**Figure 2:**
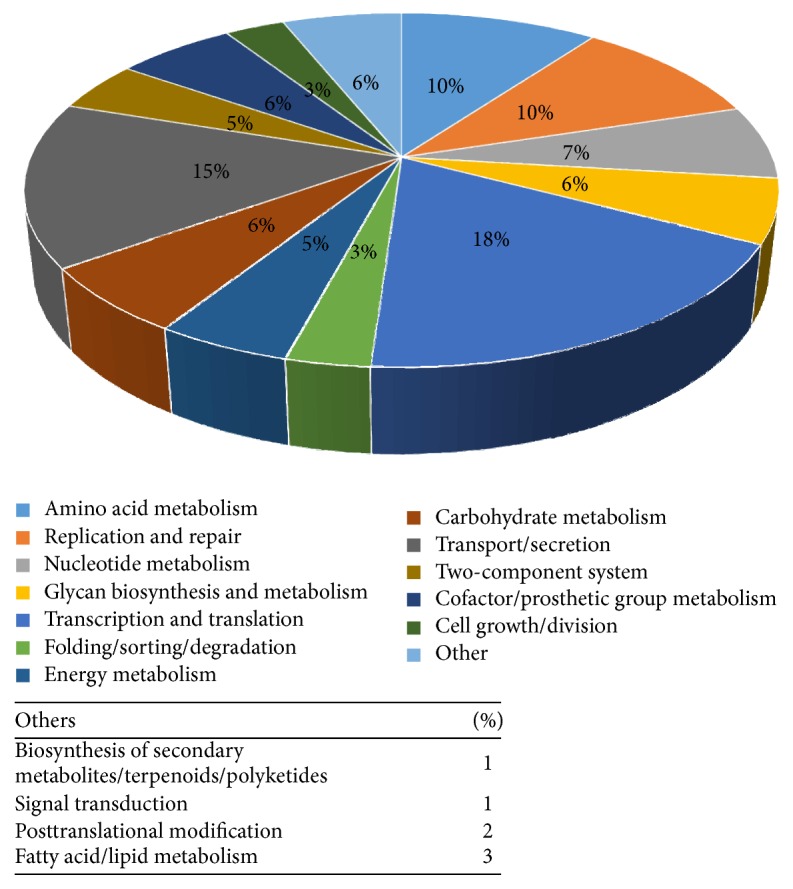
A pie-chart depicting the distribution of the 297 drug targets in* Dichelobacter nodosus* into the major metabolic pathways.

The candidate proteins were analyzed using KAAS for pathway annotation [29]. Out of 361 proteins, pathway annotation for 297 proteins was reported by the KAAS; the remaining 64 proteins had no pathway annotation information. The distribution of these 297 proteins into different metabolic pathways is depicted in Figure 2. Pathway annotation data file for the 297 drug targets is provided as Supplementary Material available online at http://dx.doi.org/10.1155/2016/7361361. The majority of the targets are involved in transcription and translations (60 proteins) and transport/secretion pathways (49 proteins), accounting for approximately 33% of the drug targets. Amino acid metabolism and replication and repair pathways each account for roughly 10% of the total drug targets. It should be noted that the 64 proteins which had no pathway annotation information are also potential drug targets that may be taken up for analysis and drug discovery studies. Hereafter, we have performed various analyses on this core set of 297 drug targets to identify subset of proteins with specific characteristics that may be relevant to specific drug development projects.

The bacterial pathways can be divided into two groups: (1) the pathways that are unique to bacteria only and are completely absent in mammalian host termed “unique bacterial pathways” and (2) the pathways that are common to both bacteria and the mammalian host termed “common pathways.” The unique bacterial pathways include 67 proteins annotated to the (i) two-component system, (ii) peptidoglycan biosynthesis, (iii) lipopolysaccharide biosynthesis, (iv) microbial metabolism in diverse environments, (v) photosynthesis, (vi) bacterial secretion systems, and (vii) D-alanine metabolism. The proteins belonging to the unique pathways are an ideal group of drug targets that are completely absent in host cell; host cell lacks the complete pathway and its associated proteins.

### 3.3. Identification of Metabolic Choke-Points in* D. nodosus*

We also performed a choke-point analysis on the list of 297 proteins to identify choke-point proteins. A reaction of metabolic network of a given organism which either consumes a specific substrate or produces a specific product is defined as a choke-point reaction [34]. The metabolite in focus should not be a final end product. Inhibiting a choke-point reaction/protein may lead to cell toxicity and death due to accumulation of an intermediate metabolite (in case of a protein utilizing a unique substrate) or due to paucity of one or more essential downstream metabolites (in case of a protein producing a unique product) [34]. Out of the 297 drug targets that were analyzed, 138 were identified as choke-point proteins. Out of the total identified choke-point proteins, 29 proteins belong to the unique pathways and the rest are part of common pathways (see Table 1).  Table 2 lists a subset of 29 choke-point proteins that belong to unique pathways in bacterial system. Proteins belonging to this subset will be (a) safer targets as the complete pathway is absent in the host and, therefore, probability of cross-interaction of drugs is further reduced, (b) druggable targets due to presence of substrate-binding pockets, which may be gainfully exploited for drug development, and (c) effective targets because inhibition of these choke-point proteins is expected to produce a blockade in the pathway which may create an unsustainable condition inside the bacterial cell. Hence, this group of proteins are predicted to be attractive candidates in their respective pathways for the design of potent inhibitors.

### 3.4. Classification Based on Predicted Subcellular Localization

**Figure 3:**
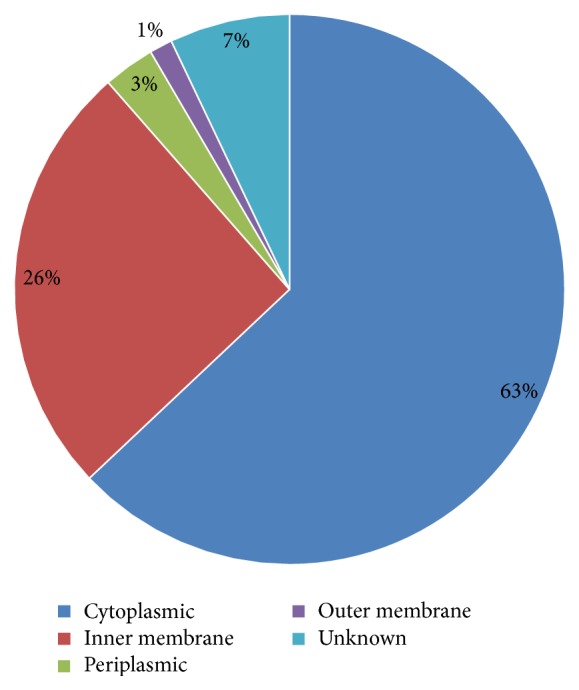
A pie-chart showing the distribution of the drug targets in* Dichelobacter nodosus* on the basis of their subcellular localization.

Determination of subcellular localization of proteins is useful in genome/proteome analysis and annotation. Especially in case of pathogenic species, knowledge of subcellular localization of proteins is particularly useful in revealing cell surface and extracellularly secreted proteins that may be involved in pathogenesis. Since these proteins are the most accessible to any form of external intervention, hence they are considered attractive vaccine as well as drug targets. The distribution of the predicted subcellular localization for the 297 putative drug targets is depicted in Figure 3 (see Supplementary Material for raw data). While none of the proteins were predicted to be extracellular, 89 were predicted to be membrane-associated proteins, out of which 76 were inner membrane-associated, 9 were periplasmic, and 4 were outer membrane-associated proteins. A total of 187 proteins were predicted to be cytoplasmic proteins and for the remaining 21 the subcellular localization was unknown. It should be noted that the absence of any predicted extracellular protein could be a consequence of the workflow employed in the present study that biases the obtained results towards cytoplasmic and membrane proteins. Since extracellular proteins are generally not essential for the survival of the pathogen, they would not appear in the list of targets identified based on a homology with known essential proteins. However, many of them may be critical for promoting pathogenicity and survival of the pathogen inside the host tissues. Such secreted effector proteins can also be attractive targets for drug as well as vaccine development. It may therefore be useful to carry out subcellular localization prediction without incorporating* a priori* essentiality criteria for shortlisting protein targets, thereby allowing the identification of pathogen-specific extracellular proteins.

The 21 protein targets for which no localization prediction was obtained could also be considered for further investigations to identify correct localization and prioritized accordingly towards drug development studies. Experimental localization studies using fluorescent tags may be performed for this set of protein targets; this would aid in uncovering novel drug targets that are specific to the pathogen of interest.

### 3.5. Identifying Drug Targets against Multiple Pathogens

**Figure 4:**
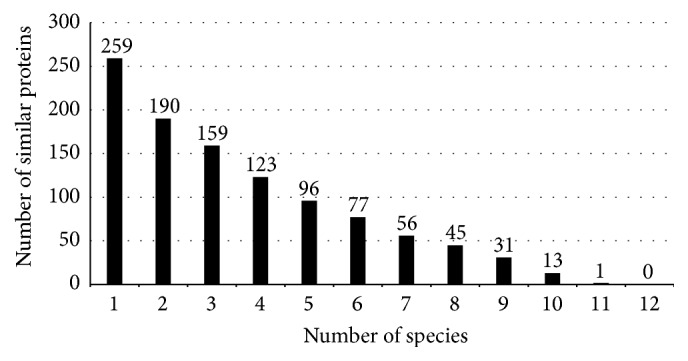
Bar-graph depicting the number of* Dichelobacter nodosus* genes that are homologous with proteins of different number of pathogenic bacterial species.

Since ovine foot rot is characterized by lesions at the hoof that are largely exposed, there is a possibility of multiple infections developing at the lesion. Proteins that are essential in multiple pathogens can be ideal drug targets for designing of broad-spectrum antibiotics that can be used for treating difficult cases of mixed infections. Therefore, we analyzed the 297 drug targets to look for conservation across 12 pathogenic bacterial species (see Section 2.5 for the list) by performing a BLASTP analysis against the DEG database for these 12 species [22]. The results of this analysis are depicted in Figure 4. Out of the 297 drug targets, we found 259 proteins to be essential and similar in at least 1 species. On the other hand, none of the proteins of* D. nodosus* were found to be essential and similar in all 12 species; only a single protein was found to have a similar match in 11 species. The 13 drug targets that were essential and conserved in at least 10 pathogenic bacterial species are tabulated in Table 3. These proteins candidates could potentially be targeted for drug development for treating infections caused by multiple pathogens and can be studied further for development of broad-spectrum antibiotics. Further, we find that, out of these 13 proteins, 5 proteins are choke-points within pathways that are unique to bacterial cells (indicated with *∗* in Table 3). These proteins include FtsI and penicillin-binding protein 2 that are targets for broad-spectrum *β*-lactam antibiotics. The other three (MurA, MurC, and MurG) are proteins that are essential for peptidoglycan biosynthesis; while MurA is already a target for fosfomycin, MurC and MurG could be explored further using computational and experimental methods as targets for design of broad-spectrum antibiotics. Computational studies may include development of homology-based protein models, virtual screening, and simulation studies of targets for drug discovery. Using sequence homology information, it is also possible to predict drug molecules that are likely to be good inhibitors of the candidate protein. For novel targets with no significant homology to available structures, crystallographic studies can be performed to aid the computational efforts for designing novel drugs.

## 4. Conclusion

*In silico* comparative genomics and bioinformatics approaches allow us to rationally narrow down the number of targets that may be considered for drug discovery workflows. We have identified a set of 361 proteins that are essential for* Dichelobacter nodosus* and are nonhomologous with the sheep proteome. The prediction of essential genes in the present study is based on the assumption that proteins homologous with known essential genes should also be essential. It is therefore recommended that, before selecting a final list of targets for drug development, experimental studies are conducted to validate the essentiality of the target proteins. Essentiality of a protein may be assayed in bacteria using conditional or temperature-sensitive mutants. From this set, 297 proteins with associated pathway annotations were examined further for subcellular localization, conservation in multiple pathogens, and so forth. Such analyses allow the identification of a specialized set of targets that are suitable for drug discovery approaches.

In summary, the present study has resulted in the generation of a list of proteins that may be considered for target-based drug discovery. In addition, the results also suggest that essentiality-based selection criteria of putative drug targets may not be suitable for detection of novel extracellular effectors of* Dichelobacter*; perhaps, consideration of this aspect will facilitate future computational studies that focus on identification of putative bacterial effector proteins. In general, the work lays down the foundation for future computational and experimental studies on the identified drug targets for design of novel drugs against ovine foot rot.

## Supplementary Material

Supplementary includes a text file that contains; a) Pathway annotation for all putative 297 drug targets as derived from KEGG Automated Annotation Server (KAAS), b) Raw output from PSORTb server which includes predicted subcellular localization of all 297 targets.

## Figures and Tables

**Table 1:** Distribution of the identified 297 putative drug targets into “unique bacteria pathways” and “common pathways.” The number of proteins that are involved in choke-point reactions within each group is also tabulated.

Pathway group	Number of proteins	Number of choke-point proteins
Unique bacterial pathways	67	29
Common pathways	230	107
Total	297	138

**Table 2:** List of 29 proteins that are part of unique bacterial pathways that are completely absent in host and are also choke-point proteins.

S. number	PID	Unique metabolic pathway and associated choke-point protein(s)	EC number
(I)	Microbial metabolism in diverse environments		

	146328927	Diaminopimelate epimerase	5.1.1.7
	146329519	Aspartate kinase	2.7.2.4
	146329782	2,3,4,5-Tetrahydropyridine-2-carboxylate N-succinyltransferase	2.3.1.117
	146329390	4-Hydroxy-tetrahydrodipicolinate reductase	1.17.1.8
	146329218	Aspartate-semialdehyde dehydrogenase	1.2.1.11

(II)	Lipopolysaccharide biosynthesis		

	146329080	UDP-2,3-diacylglucosamine hydrolase	3.6.1.54
	146329113	Heptosyltransferase I	2.4.—.—
	146328792	Heptosyltransferase II	2.4.—.—
	146328867	UDP-3-O-[3-hydroxymyristoyl] N-acetylglucosamine deacetylase	3.5.1.108
	146329045	3-Deoxy-D-manno-octulosonic-acid transferase	2.4.99.12
	146329875	Lipid A biosynthesis lauroyl acyltransferase	2.3.1.—
	146329714	2-Dehydro-3-deoxyphosphooctonate aldolase	2.5.1.55
	146329082	3-Deoxy-manno-octulosonate cytidylyltransferase	2.7.7.38
	146329066	Tetraacyldisaccharide 4′-kinase	2.7.1.130
	146328829	Lipid-A-disaccharide synthase	2.4.1.182
	146329695	UDP-N-acetylglucosamine acyltransferase	2.3.1.129

(III)	Methane metabolism		

	146328905	Phosphate acetyltransferase	2.3.1.8
	146329331	Acetate kinase	2.7.2.1

(IV)	Peptidoglycan biosynthesis		

	146328739	Penicillin-binding protein 1B	2.4.1.129
	146329826	Penicillin-binding protein 2	—.—.—.—
	146328649	Cell division protein FtsI	2.4.1.129
	146328685	UDP-muramoylpentapeptide-N-acetylglucosaminyltransferase	2.4.1.227
	146329801	UDP-N-acetylmuramoyl-tripeptide–D-alanyl-D-alanine ligase	6.3.2.10
	146329007	UDP-N-acetylmuramoyl-L-alanyl-D-glutamate–2,6-diaminopimelate ligase	6.3.2.13
	146328783	UDP-N-acetylmuramoylalanine–D-glutamate ligase	6.3.2.9
	146329426	UDP-N-acetylmuramate-alanine ligase	6.3.2.8
	146329258	Phospho-N-acetylmuramoyl-pentapeptide-transferase	2.7.8.13
	146329145	UDP-N-acetylglucosamine 1-carboxyvinyltransferase	2.5.1.7
	146328696	UDP-N-acetylmuramate dehydrogenase	1.3.1.98

**Table 3:** List of 13 proteins in *Dichelobacter nodosus* that were common in at least 10 pathogenic bacterial species. The results for the choke-point analysis for each protein are also tabulated.

S. number	Protein	KEGG ID	Choke-point protein (yes/no)
1	Cell division protein FtsI^*∗*^	K03587	Yes
2	UDP-muramoylpentapeptide beta-N-acetylglucosaminyltransferase (MurG)^*∗*^	K02563	Yes
3	30S ribosomal protein S3	K02982	No
4	UDP-N-acetylglucosamine 1-carboxyvinyltransferase (MurA)^*∗*^	K00790	Yes
5	Cell division protein FtsZ	K03531	No
6	D-Alanine-D-alanine ligase	K01921	No
7	RNA polymerase sigma-32 factor	K03089	No
8	UDP-N-acetylmuramate-alanine ligase (MurC)^*∗*^	K01924	Yes
9	Replicative DNA helicase	K02314	No
10	RNA polymerase sigma-70 factor	K03086	No
11	Transcription termination factor	K02600	No
12	Penicillin-binding protein 2^*∗*^	K05515	Yes
13	DNA polymerase III subunit alpha	K02337	No

*∗* indicates proteins that are among the 29 proteins listed in Table 2.
